# Evaluating Diagnostic and Prognostic Value of Plasma miRNA133a in Acute Chest Pain Patients Undergoing Coronary Angiography

**DOI:** 10.1097/MD.0000000000003412

**Published:** 2016-04-29

**Authors:** Jia Ke-Gang, Li Zhi-Wei, Zhang Xin, Wang Jing, Shi Ping, Han Xue-Jing, Tang Hong-Xia, Tang Xin, Liu Xiao-Cheng

**Affiliations:** From the Department of Clinical Laboratory (K-GJ, Z-WL, JW, PS, X-JH, H-XT, XT), Department of Surgery (X-CL), TEDA International Cardiovascular Hospital, Cardiovascular Clinical College of Tianjin Medical University, Tianjin, China; and Tianjin Second Hospital (XZ), Zhong Shan Road, HeBei District, Tianjin, China.

## Abstract

Circulating microRNA has recently emerged as a promising biomarker for cardiovascular disease. This study sought to evaluate the diagnostic and prognostic value of circulating miR-133a as a marker of acute myocardial infarction in acute chest pain patients undergoing coronary angiography.

Plasma was collected from 312 patients with chest pain on admission in the emergency department and 67 healthy controls. MiR-133a was detected using real-time quantitative PCR and enhanced accu-TnI, creatinine kinase-MB mass, and myoglobin were measured by immunoassay. End-point events (serious adverse cardiovascular events which require hospitalization or cardiovascular death) were examined in the AMI (acute myocardical infarction) group within 1, 6, 12, and 24 months.

The miR-133a level was higher in AMI patients than in non-AMI patients (*P* < 0.001). In the ROC analysis, the sensitivity of miR-133a in diagnosis of AMI is 0.61 and the specificity is 0.68. In the prognostic analysis, only 1 endpoint event was observed in the non-AMI group; the amount of cases with end-point events in the AMI group at 1,6,12, and 24 months were 8, 19, 28, and 35, respectively. The cutoff value of miR-133a was determined using the median value of the AMI group and separated the patients into a positive group and a negative group. The Kaplan–Meier survival curve showed no significant difference in survival was detected in AMI patients between the miR-133a positive group and negative group after follow-up (12-month: *x*2 = 1.353, *P* = 0.245; 24-month: *x*2 = 3.722, *P* = 0.054). After adjusting for age, gender, Killip classes, prior myocardiac infarction history, myoglobin, LVEF (left ventricular ejection fraction), diabetes, hypertension, smoking and systolic blood pressure, miR133a had a significant association with the risk of events at 12 months (HR = 2.869, *P* = 0.024) and 24 months (HR = 3.936, *P* = 0.001).

In patients undergoing coronary angiography, circulating miR-133a is upregulated in AMI patients, but it does not provide enough accuracy for clinical AMI diagnosis because it also rises in unstable angina patients. Its prognostic value in AMI is uncertain mainly for the number of cases with end-point event was small and may be further validated in a larger, better designed study.

## Abstract 2

Supplemental Digital Content is available in the text

## INTRODUCTION

Acute myocardial infarction is a severe cardiac disease. Early diagnosis and treatment can significantly reduce mortality and improve long-term prognosis.^1^ Therefore, identifying novel biomarkers with high sensitivity and specificity for early diagnosis of AMI is essential to improve outcomes in patients with acute chest pain. Circulating microRNA has recently emerged as a promising biomarker for cardiovascular disease.^2,3,4^

MicroRNAs (miRNAs) are a class of highly conserved, small (19–25 nucleotides) noncoding post-transcriptional regulators. Each miRNA can regulate hundreds of messenger RNA targets and participate in all biological processes, including cancer, metabolism, stem cell regulation, and immune function.^5^ Circulating miRNAs are easily detectable, relatively stable, and tissue-specific,^6^ which making them attractive as biomarker candidates. Previous studies reported that muscle-specific miR-1, miR-133a, and miR-499 as well as cardiac-specific miR-208a levels are significantly higher in plasma of AMI patients than in healthy controls, coronary heart diseases (CHD) patients without AMI, or patients with other cardiovascular diseases; the highest magnitude of change in these 4 miRNAs is miR-133a,^7,8^ As miR-133b is specifically expressed in skeletal muscle, not in the heart,^9,10^ we chose miR-133a as our candidate miRNA. Several studies have reported miR-133a as a biomarker for AMI,^11–14^ but the sample sizes of these studies are small. We compared expression of miR-133a and biomarkers such as creatinine kinase-MB (CK-MB) isoenzymes, cardiac myoglobin and troponins, which are routinely used in clinical diagnosis^15^ in acute chest pain patients to explore the differential diagnostic value of miRNAs. Biomarkers could reflect inflammation and oxidative stress.^16^ They had some potential prognosis value. Widera reported that miR-133a was significantly associated with the risk of death in ACS patients.^17^ This study sought to evaluate the diagnostic and prognostic value of circulating miR-133a as a marker of acute myocardial infarction in acute chest pain patients undergoing coronary angiography.

## METHODS

### Study Population

**FIGURE 1:**
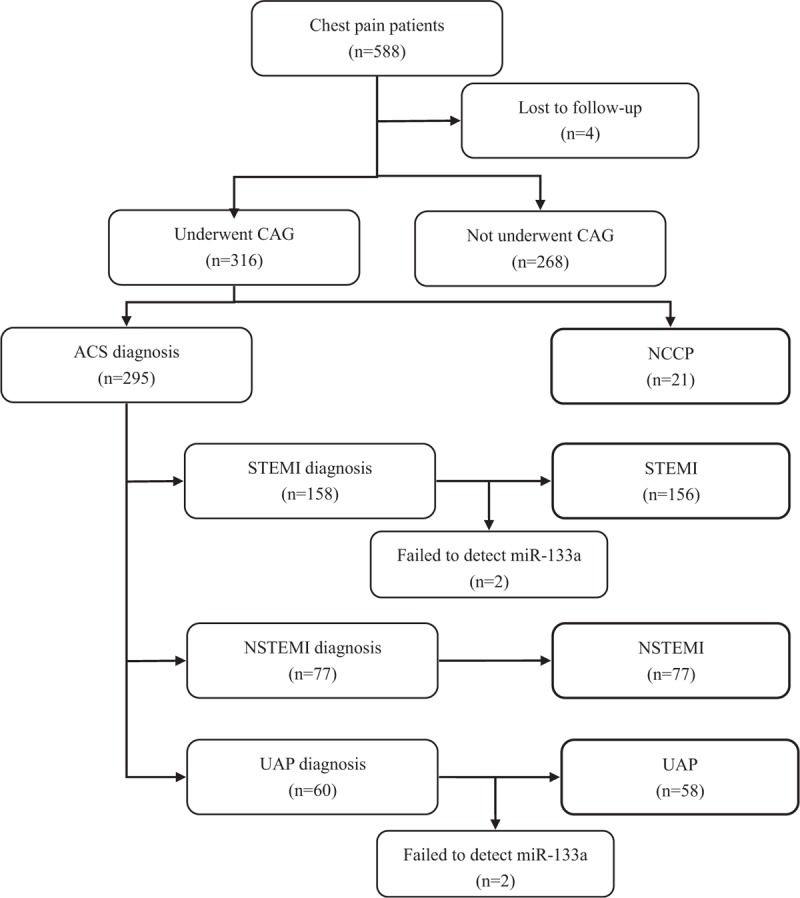
The flow diagram of participation.

We gathered blood samples from 588 chest pain patients at TEDA International Cardiovascular Hospital Emergency Department between September 2011 and August 2012. Patients with contraindications (majorly renal impairment caused by hypertensive nephropathy and diabetic nephropathy) had not undergone coronary angiography and were excluded from the study. After this, 316 patients were included in the study (Figure 1). A total of 67 healthy adults (normal electrocardiogram and no history of cardiovascular diseases) were enrolled into the study. All the patients were recruited prospectively. The diagnosis was based on the latest developed standard definition.^18^ All participants underwent clinical evaluation, including physical examination, 12-lead electrocardiagram, and echocardiographic examinations. Two experienced heart doctors who were blinded to the miR-133a concentrations determined diagnoses. Clinical data including age, sex, coronary risk factors (hypertension, diabetes mellitus, hyperlipidemia, and smoking), renal function, Killip classes, time since the onset of chest pain, treatment assignments, and diagnosis were collected from hospital medical records. The TEDA International Cardiovascular Hospital's ethics committee approved the study and each participant provided written informed consent.

### Sample Preparation

Peripheral blood was collected in tubes containing EDTA and centrifuged at 820-× g for 5 minutes at room temperature. Plasma was transferred to a fresh RNase/DNase-free Eppendorf tube and stored at −80 centigrade pending RNA extraction. All blood samples were processed within 4 hours after collection. Total plasma RNA was isolated and eluted in 100 μL of RNase-free water using a mirVana PARIS kit (#1556, Ambion) following the manufacturer's protocol for liquid samples. For individual miRNA tests, 500 μL of plasma from each participant was used. All plasma RNA preparations were quantified by Nano Drop 1000 (Thermo Scientific).

### Quantitative PCR

RNA (5 μL) was reverse-transcribed using the TaqMan microRNA Reverse Transcription kit (ABI) according to manufacturer's instructions. Subsequently, 2.33 μL of the product were used for detecting miRNA expression by quantitative qPCR using TaqMan microRNA Assay kits (ABI) for the corresponding miRNA. The primers used for quantitative real-time PCR were as follows: miR-133: forward 5′-GGGTTTGGTCCCCTTCAA-3′, reverse 5′- AGTGCGTGTCGTGGAGTC-3′; for the derivation cohort, values were normalized to cel-miR-39 and are expressed as 2- (CT[microRNA133a]-CT[cel-miR-39]).

### Biochemical Analyses

Accu-cTnI, CK-MB, and Myo were measured from blood samples with the use of chemiluminescence immunoassay (CK-MB by means of mass assay, Beckman Coulter).

### Study End Point and Follow-Up

The study end points were the occurrence of cardiovascular death, myocardial infarction, hospitalization for unstable angina, stroke, coronary revascularization procedures, peripheral revascularization procedures, or heart failure requiring hospitalization. To avoid double counting of patients with >1 event, each patient contributed only once to the composite end point. The patients′ end points or events were checked by reviewing medical records or through follow-up telephone interviews up to 24 months after the chest pain episode.

### Statistical Analysis

The data is represented as median (range) unless otherwise indicated. The quantitative data were evaluated to determine whether they followed the normal distribution by the Shapiro–Wilk test. The Levene test of homogeneity of variance was performed. When the data fitted the homogeneity of variance, independent-samples *t*-tests were applied. For the quantitative data that did not fit the normal distribution or the homogeneity of variance as well as ranked data, the Wilcoxon rank sum test was performed. The qualitative data were compared by the Pearson *x*^2^ test. The post-hoc test was performed using the same method with Bonferroni correction. The receiver operating characteristic (ROC) curves were established for discriminating chest pain patients from the control subjects. We examined each cardiac biomarker, obtained ROC curves, and the cutoff values, and then evaluated their sensitivity, specificity, positive predictive values (PPVs), and negative predictive values (NPVs). Correlations analysis was performed using the Spearman test to examine correlations between the levels of the miR-133a and cardiac biomarkers. The correlations of cardiac biomarkers with end-point events for 1, 6, 12, and 24 months of follow-up were evaluated by the Kaplan–Meier survival curve with log-rank test and multivariable Cox regression (step forward selection method based on the maximum likelihood ratio) analyses. All *P* values were 2-sided and *P* < 0.05 was considered statistically significant if not specified. Statistical analysis was performed using the statistical software SPSS 13.0 and Graphpad Prism 5. The statistical powers of the Cox regression were calculated using the software NCSS-PASS 11.

## RESULTS

### Patient Characteristics

Results for control patients are presented in Supplementary Table 1, and multivariate analyses are presented in the Supplementary Table 2 and Supplementary Table 3. Baseline characteristics of the chest pain patient population at admission to the chest-pain unit are provided in Table 1. None of the variates followed the normal distribution. The distribution of the time of chest-pain onset was similar in all diagnosis groups, and the median was 4 hours (0.5–12 hours). Variables such as age, prior MI, percutaneous coronary intervention, coronary-artery bypass grafting, smoking, hypertension, diabetes, medications, glucose (Glu), total cholesterol, low-density lipoprotein (LDL-C), LVEF, CK-MBmass, Myo, accu-cTnI, and miR-133a were statistically different among the 4 groups of chest pain patients: ST-elevated myocardial infarction (STEMI), non-ST-elevated myocardial infarction (NSTEMI), unstable angina pectoris (UAP), and noncardiac chest pain (NCCP).

**TABLE 1:**
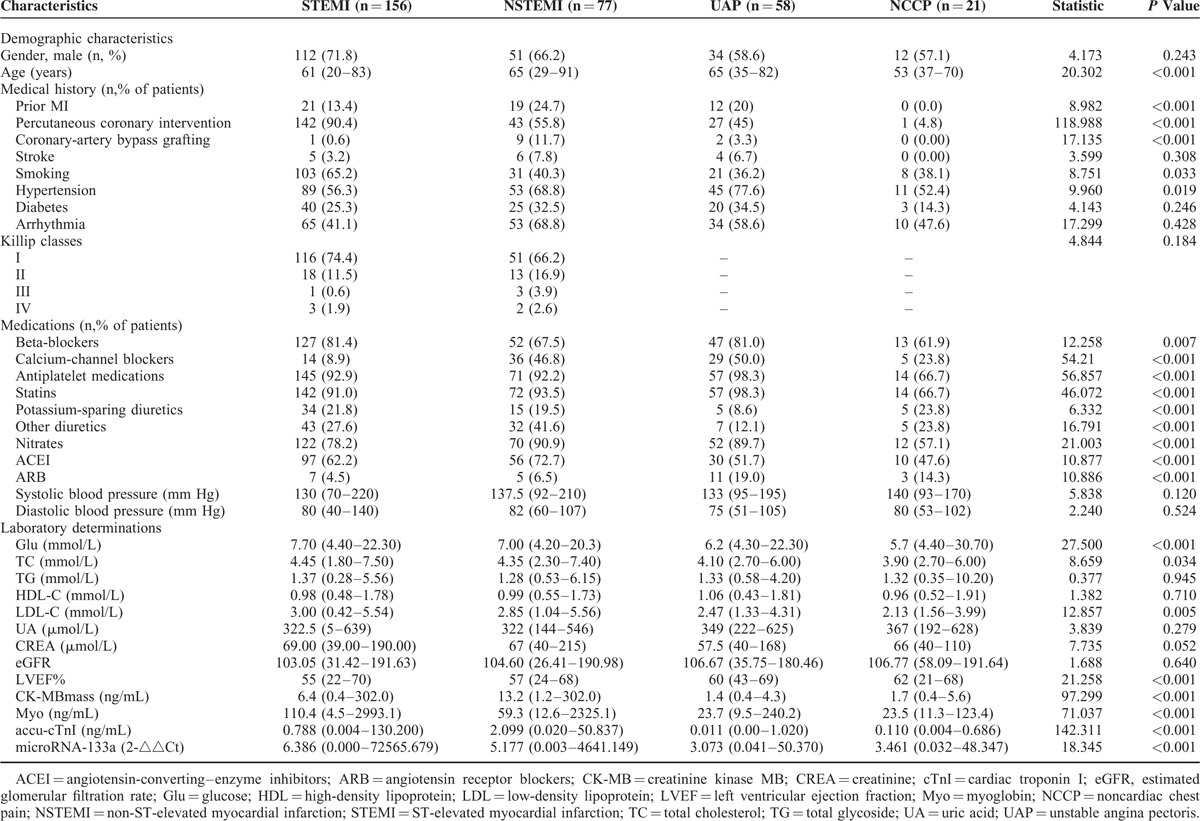
Clinical Characteristics of Chest Pain Patients

After Bonferroni correction, *P* < 0.008 was considered statistically significant. The post-hoc test showed that AMI groups have a higher Glu level than non-AMI groups; NSTEMI and UAP groups are older; the STEMI group has more PCI, higher LDL level, and lower LVEF than non-AMI groups; there are more hypertension in NSTEMI and UAP groups. The prior MI, diabetes, and all the medication information were significant different in all the 4 groups (*P* < 0.001).

### Plasma Levels of miR-133a in Chest Pain Patients and Healthy Controls

**FIGURE 2:**
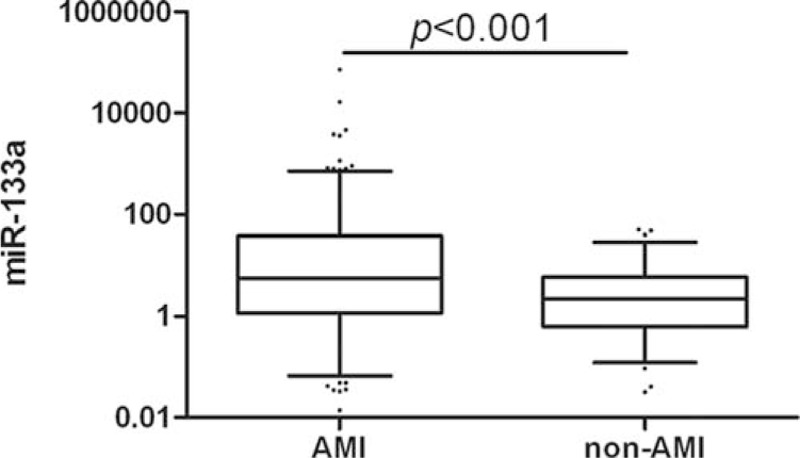
Relative expression of miR-133a in plasma of AMI versus non-AMI.

**FIGURE 3:**
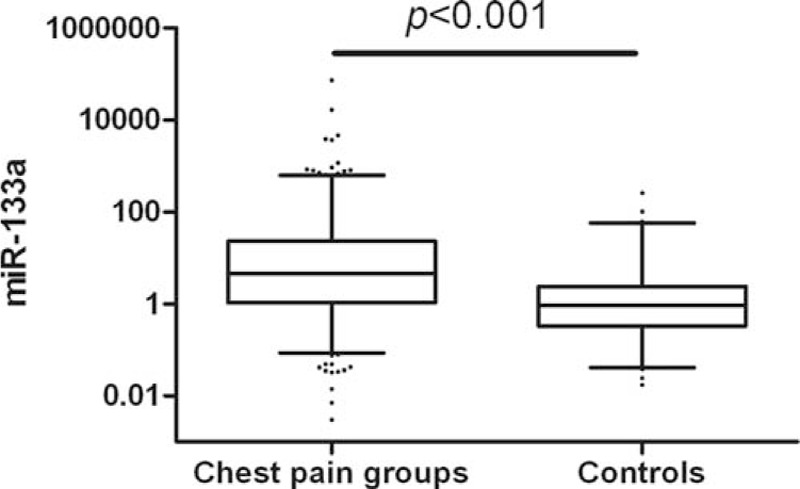
Relative expression of miR-133a in plasma of chest pain patients versus healthy controls.

**FIGURE 4:**
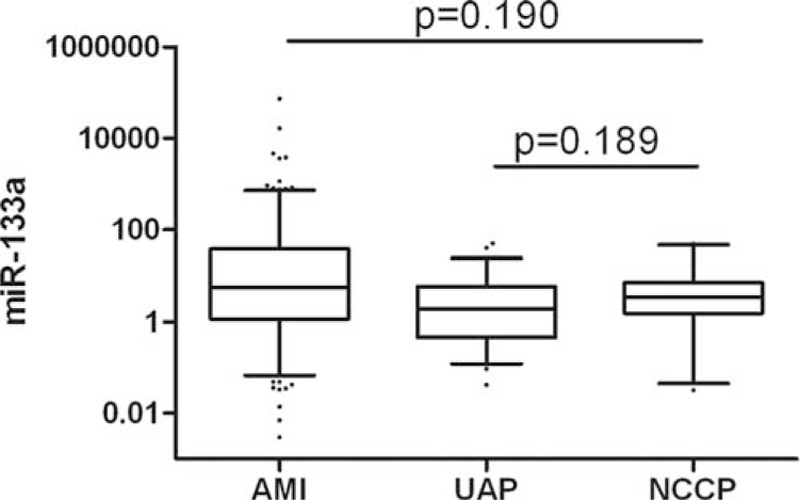
Relative expression of miR-133a in plasma of AMI and UAP versus NCCP.

Relative expression of miR-133a, normalized to cel-miR-39, was analyzed by qRT-PCR in all chest pain patients and healthy controls. Plasma levels of miR-133a was higher in AMI patients than non-AMI patients (*P* < 0.001, Figure 2). Plasma levels of miR-133a were 4.720-fold higher in chest pain patients than in healthy controls (*P* < 0.001, Figure 3). Further, plasma levels of miR-133a in chest pain patients varied by subgroup: miR-133a in STEMI, NSTEMI, UAP, and NCCP patients was 6.386, 5.177, 3.073, and 3.461-fold higher, respectively, than in healthy controls (Supplementary Figure 1). miR-133a expression in each subgroup of chest pain patients was statistically significantly different to that of controls; but no statistical differences was found between ACS (acute coronary syndrome) and NCCP (Figure 4 and Supplementary Figure 2).

### Specificity and Sensitivity of miR-133a as a Diagnostic Biomarker

**FIGURE 5:**
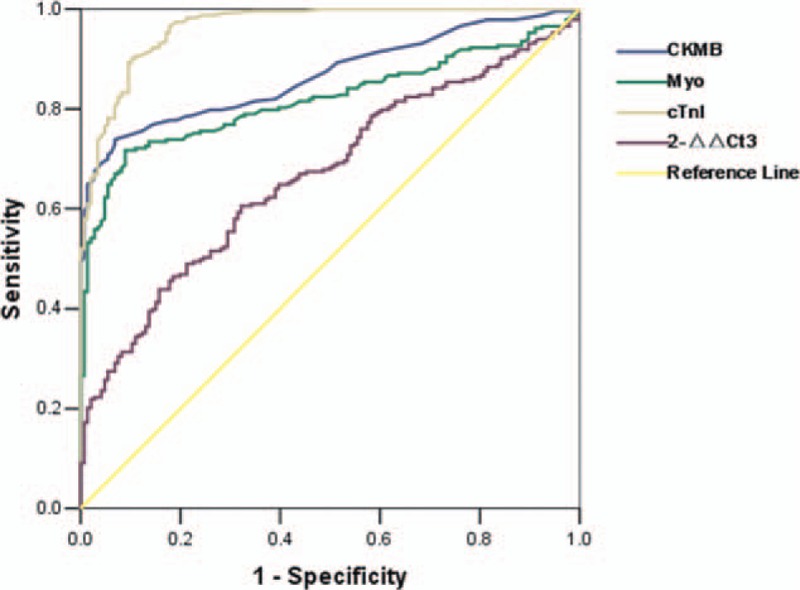
Receiver operating characteristics (ROC) curve analysis of miR-133a, accu-cTnI, CK-MB, myoglobin in the AMI group (STEMI, NSTEMI) versus non-AMI group (UAP, NCCP, controls) on admission, *P* < 0.001.

Next, we performed ROC analysis in AMI (STEMI, NSTEMI) patients versus non-AMI (UAP, NCCP, Health Controls) population to examine whether circulating miR-133a could be used as a diagnostic biomarker for myocardial infarction. Figure 5 shows the ROC analysis of CK-MB, Myo, accu-cTnI, and miR-133a. The sensitivity of miR-133a in diagnosis of AMI is 0.61 and the specificity is 0.68 (Table 2). Areas under the curve of these markers were 0.867, 0.819, 0.964, and 0.667, respectively (Figure 5, Table 2); and cutoff values of CMKB, Myo, accucTnI, and miR-133a were 2.95, 38.45, 0.0435, and 3.5665, respectively.

**TABLE 2:**
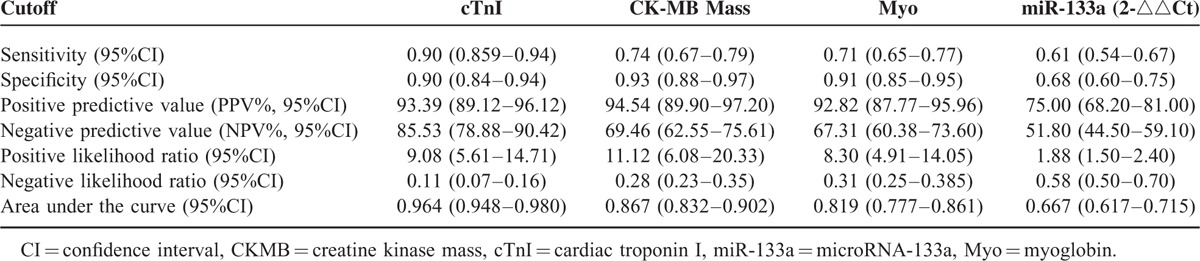
Cardiac Biomarkers and Their Diagnostic Validity for Myocardial Infarction

### Kaplan–Meier Survival Curve of End-Point Events

**FIGURE 6:**
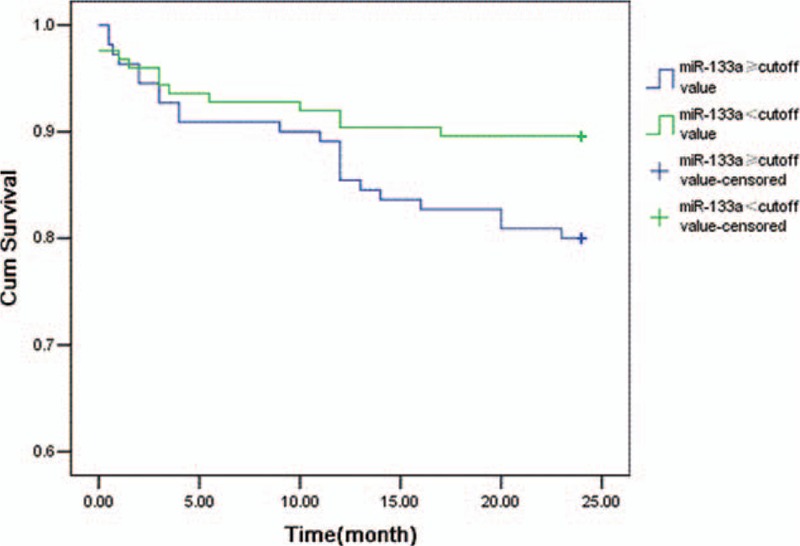
Kaplan–Meier survival curves for 24 months to end-point events in patients with or without AMI.

To further investigate the utility of miR-133a as a potential biomarker, Kaplan–Meier analysis was performed on data for patients with AMI (The non-AMI group was not included in the analysis because only 1 endpoint event was observed in the non-AMI group). The amount of cases with end-point events at 1,6,12, and 24 months were 8, 19, 28, and 35, respectively. We determined the cutoff value of miR-133a using the median value (not the cutoff value of the ROC curve because its diagnostic value is limited) of the AMI group and separated the patients into a positive group (above or equal to the cutoff point) and a negative group (below the cutoff point). At the 24-month follow-up, the Kaplan–Meier curve suggested the positive group's cumulative survival rate is not significantly different from the negative group (χ2 = 3.722, *P* = 0.054, Figure 6). No significant association was found at the 1, 6, or 12 months follow-up (Supplemental Figure 3).

### Circulating miR-133a Expression and Long-Term Prognostic Value for Acute Myocardial Infarction Cox Regression

We applied Cox regression analysis on the risk factors of the prognosis of myocardial infarction patients. In the first step, we used the forward selection method based on maximum likelihood ratio, and Killip classes, LVEF, Myo, prior MI, miR133a selected. Then some important clinical variables including age, gender, hypertension diabetes, and smoking were forced into the multivariate model. After adjusted for these covariates, circulating miR-133a level had a significant association with the risk of end-point events at 12 and 24 months (Table 3, Supplementary Table 2), but with no significant association at the 1- or 6-month follow-up (Supplemental Table 3).

**TABLE 3:**
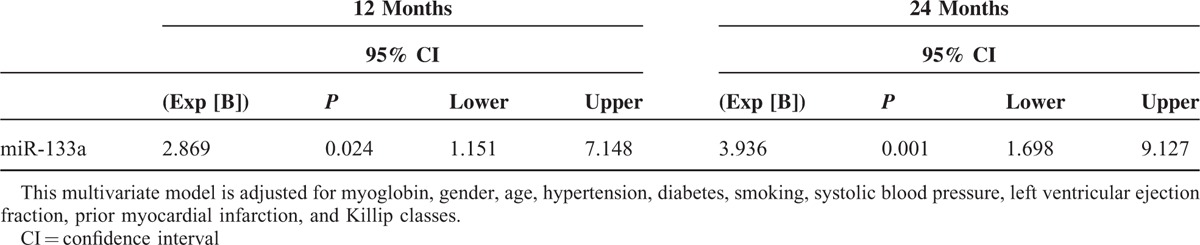
Cox Regression Analyses for End-Point Events at Follow-Up (12 and 24 Months) in Myocardial Infarction Patients

## DISCUSSION

Our study demonstrates that miR-133a is upregulated in AMI patients. It also rises in UAP patients. This indicated that it is associated with ACS.

Numerous studies have examined whether miR-133a may be an important marker for patients with acute chest pain. The results of these have been conflicted. Several studies support it as a biomarker.^11–14,19^ But the sample sizes of these studies were limited: only 305 AMI patients in total. Some of these studies only compared AMI patients with healthy controls. Our data does not support miR-133a as a biomarker, for the AUC of ROC curve is just 0.667, not enough to be used as a biomarker for AMI diagnosis. Widera reported there was a large overlap between patients with unstable angina or myocardial infarction in the microRNAs level.^17^ This means it could not distinguish between AMI patients and UAP patients. Devaux reported miR-133a does not have a diagnostic value in suspected AMI patients with an AUC of 0.53 in the ROC curve.^20^ Kuwabara reported serum levels of miR-133a increased significantly in patients not only with acute myocardial infarction but also with unstable angina pectoris and Takotsubo cardiomyopathy.^21^ These studies have a larger sample size than studies referred above which support miR-133a as a diagnostic biomarker. These studies indicate miR-133a could not be used as a diagnostic biomarker for AMI and our study confirmed this conclusion.

We also evaluated miR-133a as a prognostic predictor for end-points events in AMI patients. In univariate survival analysis, levels of miR-133a were not significantly associated with the risk of end-point events at 24 months, and in the multivariate model, there were higher relative risk in patients with a high level of miR-133a at 12 and 24 months. Using the median value of miR-133a level in AMI patients as a cutoff value, patients were separated into a positive group (above or equal to the cutoff point) and a negative group (below the cutoff point). The positive group was identified to have lower cumulative survival at 12 and 24 months, but no significant associations were detected at 1 or 6-month follow-ups.

MiR-133a plays an important role in the regulation of cardiac muscle proliferation,^22^ cardiac hypertrophy^23^ and have relationship to other injures such as myocardial ischemia-reperfusion.^24^ These factors may have some relevance to the long-term risk of AMI which requires further study to validate. One would notice that the KM curve is crossing which violate the assumption of proportional hazards. This indicates that miR-133a may be a protection factor of AMI in short term.^25^ miR-1 and miR-133a have been shown to regulate cardiomyocyte apoptosis through different mechanisms.^26,27^ This might point toward a cardio-protective role for miR-133a in the setting of myocardial infarction. This study could not give a definite conclusion due to the inadequate sample size. These questions need to be addressed thoroughly in future studies.

Elucidating the possible biological role of miR-133a in circulation after chest pain is beyond the scope of this paper. However, considering the recent reports describing cell-to-cell transport of miRNAs^28^ and of miRNAs as paracrine signaling molecules,^29^ 1 might speculate that the presence of miRNAs in circulation is not merely a by-product of myocardial necrosis but also implies a functional role for these molecules. It is interesting to notice that the change of microRNA in tissue is inconsistent with that of in circulation.^2^ We are still unclear about the mechanism of how miRNAs were released into circulation. Kuwabara reported that miR-133a levels were very low in infarcted and peri-infarcted myocardium, whereas circulation miR-133a levels increases.^22^ This study indicates that miR-133a may release into circulation from myocardium when MI occurs. Several limitations exist for our study. This study represents a single-center experience. In this study, the time required to perform a quantitative miRNA-133a assay was a minimum of 2 hours; thus, it remains impractical for an emergency situation. The need for rapid detection of the miRNA will require further research into assay techniques. Lusi et al^30^ reported an innovative simple, fast approach to detect microRNAs. As technology advances, this would not be a big problem in the future. We only measured miR-133a concentrations upon emergency department admission and the time of the onset of chest pain was not taken into account. We do not know when miR-133a is elevated, when to peak value, and when return to normal. Maybe the window phase of miR-133a is very short, and we missed the peak value time. Kidney function plays a role in the clearance of many biomarkers, but we did not systematically assess the area of myocardial injury. We did not enrol non-ACS patients. Our target population is patients who need to distinguish from AMI and acute chest pain is the most common symptom of AMI. Usually we do not screening AMI biomarkers in patients without evidence of ischemia. So we only selected ACS patients. We should not exclude patients who had not received coronary angiography (CAG). Patients underwent CAG tend to be more severe. This caused the proportion of AMI patients relatively high, and the number of non-AMI patients in our study relatively too small. Some AMI patients had not received CAG. Some patients’ renal function is not suited for CAG. This may be a selection bias.

A formal sample size calculation was not performed in our study. For the diagnostic value, from the ROC curve we could see the diagnostic accuracy of miR-133a is not so good. This is mainly because there was a large overlap between patients with unstable angina or myocardial infarction in the microRNA level. It is unlikely to eliminate this overlap by the increase the sample size; for the prognostic value, the prognostic value was evaluated by Cox regression. In the model, the power for 1, 6, 12, and 24 months were 0.453, 322, 0.759, and 0.972, respectively. The power values for 1, 6, and 12 months were not big enough. This may lead to false negative result. The sample size for 1 month and 6 months is not enough. The prognostic value of miR-133a is still uncertain.

The statistical shows miR-133a may be used as a prognostic biomarker for cardiovascular events in AMI patients. But the prognostic value is uncertain for many factors. First, plasma miR-133a was not detected in the follow-up period. If the long-term expression of miR-133a is continues higher in the high risk group, we can confirm miR-133a is significantly associated with the risk of end-point events. In my opinion, if a biomarker is associated with the risk of cardiovascular event, then either it could reflect the severity of disease or it is associated with the aggravation process. We stated in the above that miR-133a plays role in cardiac muscle proliferation, cardiac hypertrophy. This is a persistent long-term effect. Suppose miR-133a affected the cardiovascular events risk in this way, then it should persistent keep high level in plasma. We should have detected the miR-133a level in the follow-up process. Second, the severity of AMI was not fully assessed. We only assessed the Killip classes. Other factors such as TIMI risk index, number of narrowed coronary arteries, infarction size should also be taken into account. We had not collected this information. These would largely affect the result of the Cox regression. Third, medical care which patients received may be different. Medications which patients received outside of hospital are hard to control. This may have considerable influence on the prognosis especially in long-term follow-up. Finally, we may have missed some cases of fatal cardiovascular events occurred outside of hospital. These were not counted as end-point events.

## CONCLUSIONS

Whether miR-133a could be used as a biomarker for patients with acute chest pain is still controversial. Our study shows that in patients undergoing coronary angiography circulating, miR-133a is elevated in AMI patients and it could not be used as a diagnostic biomarker for AMI because it also rises in unstable angina patients. Our study shows that circulating miR-133a is significantly relevant to acute coronary syndrome. The biological mechanism is still unclear, and we think it may have some potential usage in the future.

Due to the small sample size, population selection and some other factors, the prognostic value of miR-133a is uncertain. We think the miR-133a level may predict cardiovascular events risk for AMI patients, which requires further validation in a larger, better designed study.

## Supplementary Material

Supplemental Digital Content
