# Supplementary material for: Evaluating Diagnostic and Prognostic Value of Plasma miRNA133a in Acute Chest Pain Patients Undergoing Coronary Angiography
Source: Medicine (Baltimore). 2016 Apr 29;95(17):e3412. doi: 10.1097/MD.0000000000003412 (PMC4998688; doi:10.1097/MD.0000000000003412)
Supplement: Supplemental Digital Content [file medi-95-e3412-s001.doc]

# Supplementary Figure 1


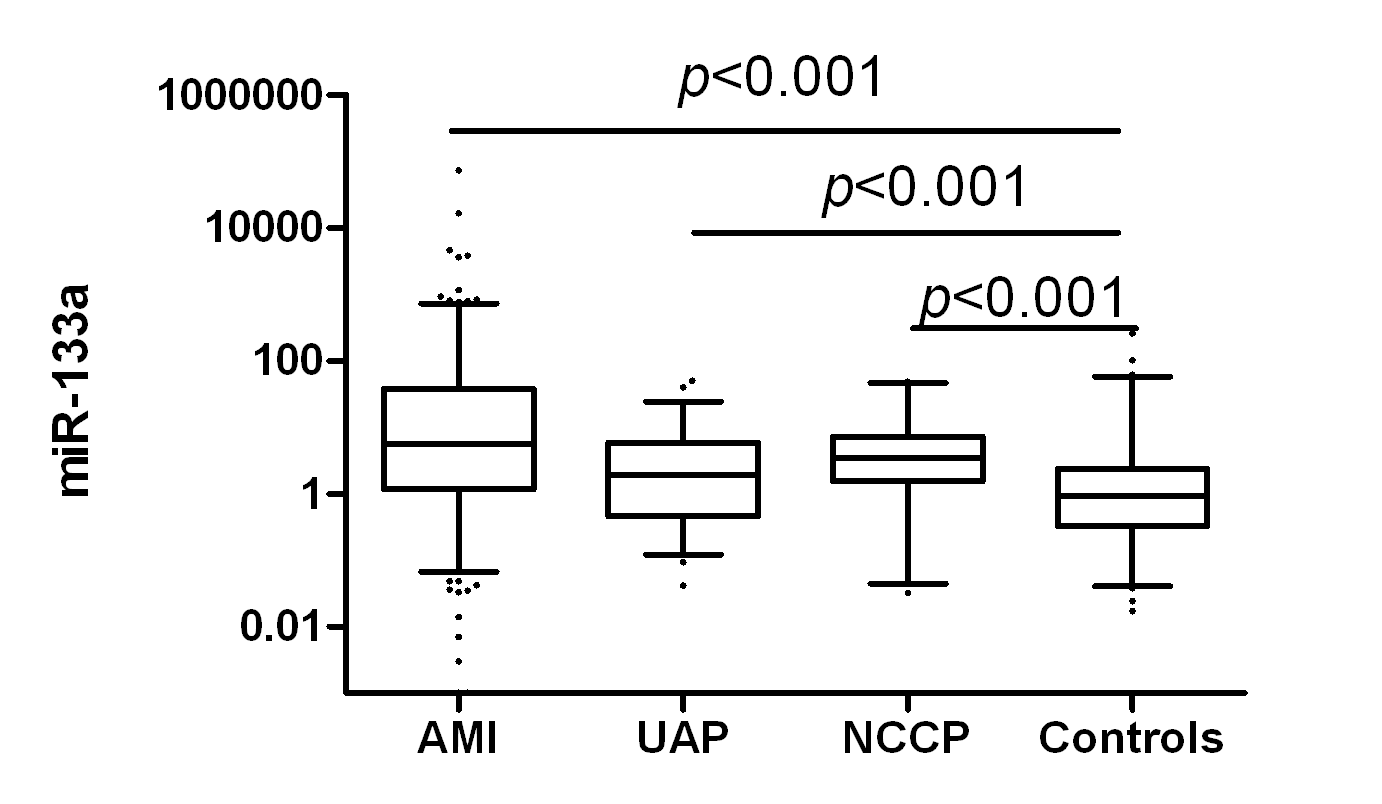


Supplementary Figure 2


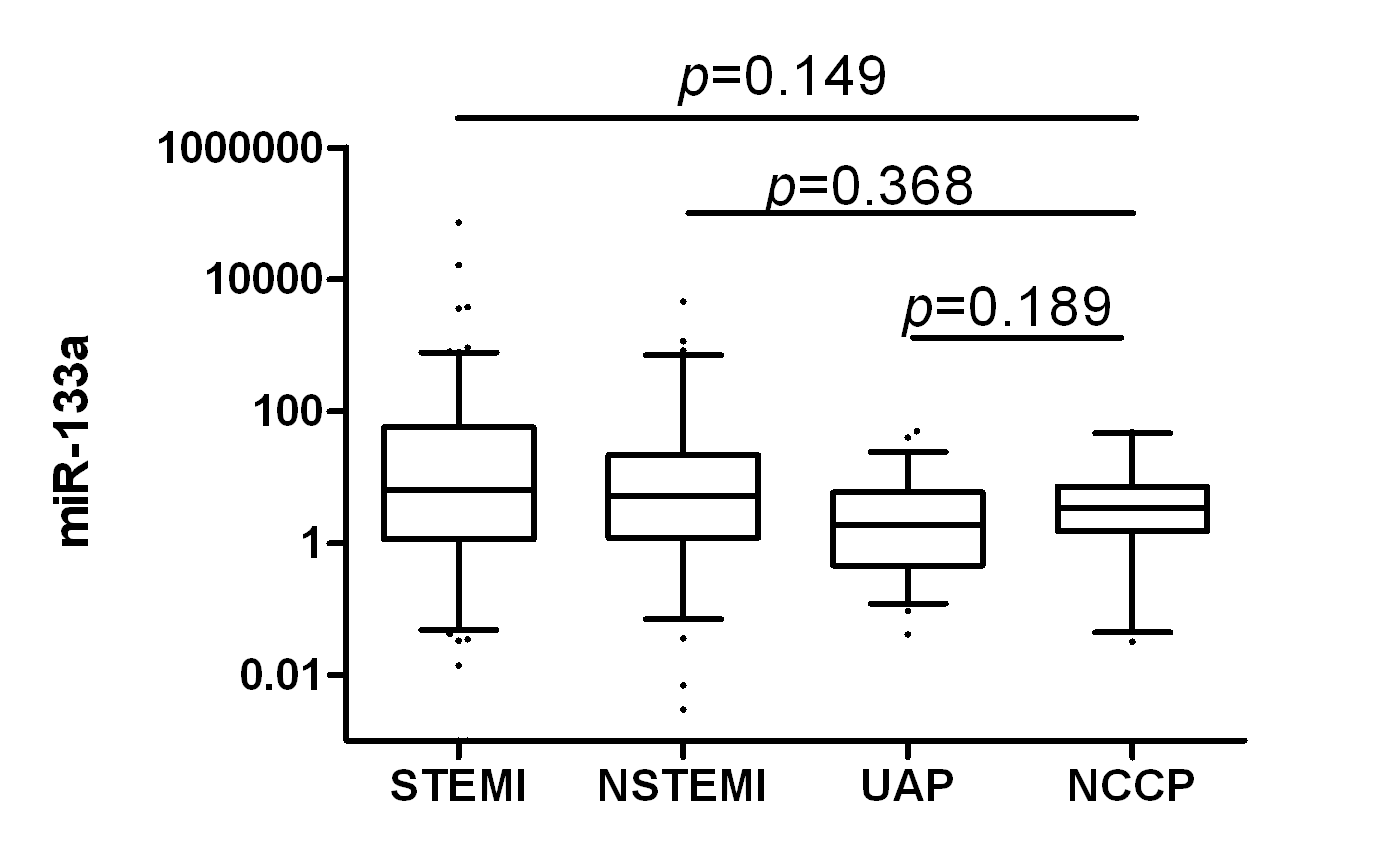


Supplementary Figure 3A


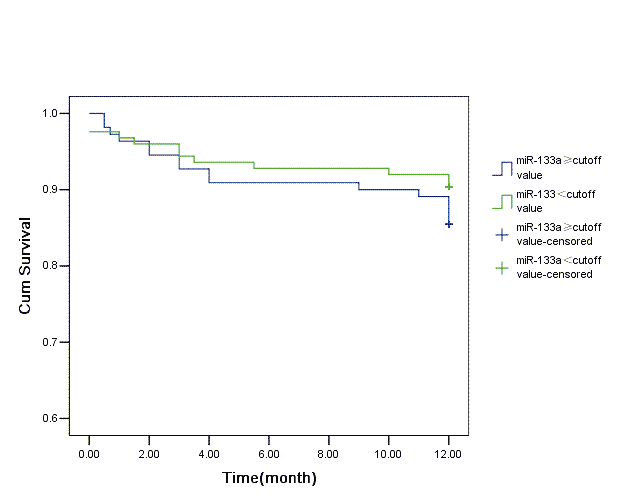


Supplementary Figure 3B


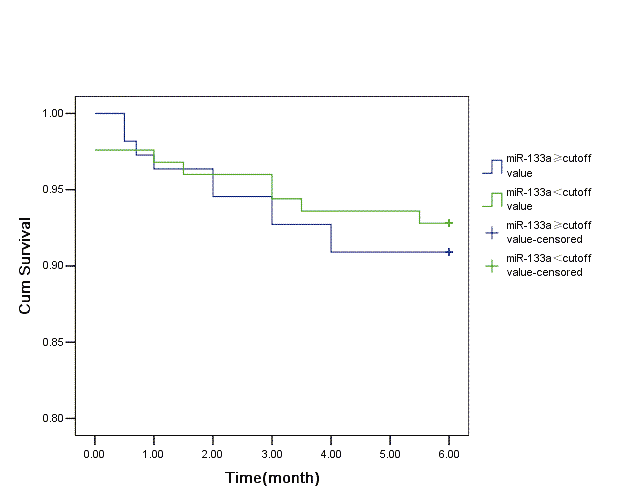


Supplementary Table 1. Clinical characteristics of the healthy patients control group

| **Characteristics** | **Controls(n=67)** |
| --- | --- |
| Gender, male (n, %) | 42(62.7) |
| Age (years) | 41(22-68) |
| Hypertension (n,%) | 0(0.00) |
| Diabetes(n,%) | 0(0.00) |
| Systolic blood pressure (mmHg) | 119.5(90-160) |
| Diastolic blood pressure (mmHg) | 80(59-110) |
| Glu(mmol/L) | 5(4-12.5) |
| ALT(U/L) | 16(6-132) |
| TC(mmol/L) | 4.7(0.6-6.8) |
| TG(mmol/L) | 1.1(0.37-3.67) |
| HDL-C(mmol/L) | 1.27(0.77-2.19) |
| LDL-C(mmol/L) | 2.92(1.47-4.37) |
| UA(umol/L) | 308.5(171-519) |
| CREA(umol/L) | 64.5(40-96) |
| cTnI(ng/ml) | 0.00(0.00-0.01) |
| microRNA-133a(△Ct) | 9.457(1.323-15.215) |

Data are presented as medians (range) for continuous measures, and N’s and percentages for categorical variables. Glu = glucose; ALT = Glutamic-pyruvic Transaminase Enzyme; TC = Total Cholesterol; TG = Triglyceride; HDL = High-density Lipoprotein, LDL = Low-density Lipoprotein, UA = Uric acid, CREA = Creatinine, CK-MB mass = Creatine Kinase-MB mass, Myo = Myoglobin, cTn I = Cardiac Troponin I.

**Supplementary Table 2. Cox regression analyses for end-point events for 12 and 24months follow-ups. Circulating miR-133a expression level suits for as a potential long-term prognosis predictor of acute** myocardial infarction.

|  | 12month | | | | 24 months | | | |
| --- | --- | --- | --- | --- | --- | --- | --- | --- |
|  | *[Exp(B)]* | *P* | *95% CI* | | *[Exp(B)]* | *P* | *95% CI* | |
|  |  |  | *Lower* | *Upper* |  |  | *Lower* | *Upper* |
| Gender | 0.896 | 0.823 | 0.342 | 2.349 | 1.392 | 0.459 | 0.580 | 3.338 |
| Age | 0.978 | 0.197 | 0.944 | 1.012 | 0.988 | 0.444 | 0.959 | 1.019 |
| Hypertension | 0.636 | 0.274 | 0.282 | 1.43 | 0.695 | 0.325 | 0.336 | 1.436 |
| Diabetes | 4.136 | 0.002 | 1.711 | 9.997 | 3.165 | 0.003 | 1.475 | 6.792 |
| Smoking | 1.158 | 0.685 | 0.569 | 2.355 | 1.091 | 0.782 | 0.587 | 2.029 |
| Myo | 1.001 | 0.000 | 1.001 | 1.002 | 1.001 | 0.001 | 1.000 | 1.002 |
| SBP | 0.979 | 0.020 | 0.962 | 0.997 | 0.980 | 0.012 | 0.965 | 0.996 |
| LVEF | 0.935 | 0.000 | 0.900 | 0.970 | 0.938 | 0.000 | 0.908 | 0.970 |
| Prior MI | 3.873 | 0.003 | 1.601 | 9.369 | 3.168 | 0.005 | 1.408 | 7.128 |
| Killip classes | 2.818 | 0.000 | 1.801 | 4.411 | 2.585 | 0.000 | 1.713 | 3.902 |
| miR133a | 2.869 | 0.024 | 1.151 | 7.148 | 3.936 | 0.001 | 1.698 | 9.127 |

**Supplementary Table 3. Cox regression analyses for end-point events for 1 and 6 month follow-ups. Circulating miR-133a expression level dose not suit for as a potential short-term prognosis predictor of acute myocardial infarction.**

|  | 1month | | | | 6 months | | | |
| --- | --- | --- | --- | --- | --- | --- | --- | --- |
|  | *[Exp(B)]* | *P* | *95% CI* | | *[Exp(B)]* | *P* | *95% CI* | |
|  |  |  | *Lower* | *Upper* |  |  | *Lower* | *Upper* |
| Gender | 0.255 | 0.286 | 0.021 | 3.131 | 0.823 | 0.963 | 0.238 | 2.867 |
| Age | 0.935 | 0.100 | 0.862 | 1.013 | 0.983 | 0.478 | 0.939 | 1.030 |
| Hypertension | 1.158 | 0.864 | 0.217 | 6.192 | 0.956 | 0.931 | 0.344 | 2.654 |
| Diabetes | 4.998 | 0.088 | 0.785 | 31.823 | 4.727 | 0.005 | 1.591 | 14.047 |
| Smoking | 7.489 | 0.060 | 0.916 | 61.223 | 2.517 | 0.048 | 1.008 | 6.288 |
| Myo | 1.000 | 0.736 | 0.998 | 1.002 | 1.001 | 0.000 | 1.001 | 1.002 |
| SBP | 0.949 | 0.031 | 0.906 | 0.995 | 0.969 | 0.009 | 0.947 | 0.992 |
| LVEF | 0.896 | 0.008 | 0.826 | 0.972 | 0.933 | 0.006 | 0.888 | 0.980 |
| Prior MI | 12.276 | 0.021 | 1.459 | 103.262 | 5.685 | 0.001 | 1.948 | 16.587 |
| Killip classes | 3.139 | 0.010 | 1.313 | 7.508 | 2.290 | 0.005 | 1.289 | 4.069 |
| miR133a | 5.041 | 0.122 | 0.650 | 39.122 | 2.119 | 0.175 | 0.715 | 6.275 |
